# Supplementary material for: NIMA-related kinase 1 (NEK1) regulates meiosis I spindle assembly by altering the balance between α-Adducin and Myosin X
Source: PLoS One. 2017 Oct 5;12(10):e0185780. doi: 10.1371/journal.pone.0185780 (PMC5628868; doi:10.1371/journal.pone.0185780)
Supplement: S1 Table — (DOCX) [file pone.0185780.s001.docx]

|  | Mean | SD |
| --- | --- | --- |
| myosin light chain 1/3, skeletal muscle isoform isoform 1f | 1.234777778 | 0.583235325 |
| myosin light chain 4 | 0.916222222 | 0.189561849 |
| myosin light chain 6B | 0.945222222 | 0.221693921 |
| myosin light chain kinase 3 isoform 1 | 1.277333333 | 0.550780129 |
| myosin light chain kinase, smooth muscle | 1.284555556 | 0.30677317 |
| myosin light chain, regulatory B-like | 1.005888889 | 0.193254654 |
| myosin light polypeptide 6 | 1.195333333 | 0.296617178 |
| myosin regulatory light polypeptide 9 | 1.086 | 0.287876276 |
| myosin-10 | 1.061222222 | 0.102613081 |
| myosin-11 isoform 2 | 1.110444444 | 0.408137266 |
| myosin-14 isoform 3 | 1.212333333 | 0.480961017 |
| myosin-7 | 1.131222222 | 0.285432031 |
| myosin-9 | 1.218111111 | 0.627280528 |
| myotubularin-related protein 1 | 1.04275 | 0.056646145 |
| myotubularin-related protein 5 isoform 1 | 1.023222222 | 0.159993576 |
| myotubularin-related protein 6 | 1.006888889 | 0.126838918 |
| myotubularin-related protein 9 | 0.910888889 | 0.144002122 |
| PREDICTED: myosin phosphatase Rho-interacting protein isoform X8 | 0.897777778 | 0.176093567 |
| PREDICTED: unconventional myosin-XVIIIa isoform X17 | 0.999888889 | 0.066242819 |
| unconventional myosin-Id | 1.199888889 | 0.353706957 |
| unconventional myosin-IXb isoform 3 | 1.149666667 | 0.447813856 |
| unconventional myosin-Va | 1.24 | 0.353951974 |
| unconventional myosin-Vb | 1.636333333 | 0.528680669 |
| unconventional myosin-VI | 1.457222222 | 0.338891862 |
| unconventional myosin-VIIa isoform 2 | 1.535 | 0.16826913 |
| unconventional myosin-X | 1.810222222 | 0.38393319 |
| unconventional myosin-XV isoform 1 | 1.914 | 0.490053313 |
| alpha-adducin isoform 1 | 0.605111111 | 0.052415276 |

**S1 Table. Myosin proteins quantitation by mass spectrometry**
